# Engineering Multifunctional Hydrogel With Osteogenic Capacity for Critical-Size Segmental Bone Defect Repair

**DOI:** 10.3389/fbioe.2022.899457

**Published:** 2022-05-09

**Authors:** Shaowei Zheng, Haobo Zhong, Hao Cheng, Xu Li, Guowei Zeng, Tianyu Chen, Yucong Zou, Weile Liu, Chunhan Sun

**Affiliations:** ^1^ Department of Orthopaedic, Huizhou First Hospital, Guangdong Medical University, Huizhou, China; ^2^ Department of Orthopaedic Surgery, Nanfang Hospital, Southern Medical University, Guangzhou, China; ^3^ Graduate School, Guangdong Medical University, Zhanjiang, China; ^4^ Department of Orthopedics, The Third Affiliated Hospital of Southern Medical University, Guangzhou, China

**Keywords:** critical bone defect, hydrogel, BMSCs, osteogenic differentiation, bone regeneration

## Abstract

Treating critical-size segmental bone defects is an arduous challenge in clinical work. Preparation of bone graft substitutes with notable osteoinductive properties is a feasible strategy for critical-size bone defects. Herein, a biocompatible hydrogel was designed by dynamic supramolecular assembly of polyvinyl alcohol (PVA), sodium tetraborate (Na_2_B_4_O_7_), and tetraethyl orthosilicate (TEOS). The characteristics of the supramolecular hydrogel were evaluated by rheological analysis, swelling ratio, degradation experiments, and scanning electron microscopy (SEM). In *in vitro* experiments, this TEOS-hydrogel had self-healing property, low swelling rate, degradability, good biocompatibility, and induced osteogenic differentiation of bone marrow mesenchymal stem cells (BMSCs) by upregulating the expression of Runx-2, Col-1, OCN, and osteopontin (OPN). In segmental bone defect rabbit models, the TEOS-containing hydrogel accelerated bone regeneration, thus restoring the continuity of bone and recanalization of the medullary cavity. The abovementioned results demonstrated that this TEOS-hydrogel has the potential to realize bone healing in critical-size segmental bone defects.

## Introduction

The treatment of critical-size segmental bone defects caused by high-energy trauma, infection, revision arthroplasty, and after resection of bone tumor is one of the most challenging clinical issues, especially for patients with poor osteogenic capability (Zhang et al., 2021; Li et al., 2022). Although autologous bone tissue possesses a certain self-healing capacity, it may still be insufficient under some special states such as critical-size segmental bone defects (Russow et al., 2019). Once critical-size defects occur, poorly healed bone tissues are easily found in some serious complications, for example, non-union, bone atrophy, bone deformities, and so on (Brandi, 2013; Bottai et al., 2015). Currently, conventional treatments like bone autografts and allografts and bone cement reconstruction have been used to repair bone defects with primary stability and long-term regeneration; however, these strategies also show some drawbacks (Bai et al., 2020b; Butscheidt et al., 2021; Chen et al., 2021). Among them, bone autografts are considered the gold standard for treatment of bone defects owing to the excellent osteoconduction, osteoinduction, and osteogenesis in bone regeneration (Hindiskere et al., 2020). However, some disadvantages, such as donor-site morbidity, high cost, and long rehabilitation time, limit the application of bone autografts (Boriani et al., 2017; Fahmy-Garcia et al., 2018; Lim et al., 2019; Han et al., 2020). Bone allografts maintain osteoconductivity, but the risks of immune rejection and infection result in inferior healing and high costs increase the economic burden, limiting its popularization (Cao et al., 2016; Santos et al., 2019). As a synthetic alternative material for bone tissue, bone cements have the opportunity to cause serious complications after implantation, for instance, non-biodegradation, exothermic reaction, the release of cytotoxic monomers, and pulmonary embolism (Lewis et al., 2010; Lai et al., 2013; Hwa et al., 2021; Nonoyama et al., 2021). Therefore, the treatment strategy which can promote critical-size bone defects healing efficiently and safely is of high clinical significance to address these problems.

Alternative bioactive materials with notable osteogenic induction effects have attracted extensive interest in bone regeneration. Recently, TEOS, a silicon-based organic compound, was used as a bone induction factor to induce osteogenesis and osseointegration at the prosthesis interface (Qiao S. et al., 2020). A series of studies have demonstrated that silicon supplementation has a significant connection with bone mineral density (Jugdaohsingh, 2007; Casarrubios et al., 2020). The silicon-incorporated polymers or cements have been explored to a great extent as materials applied in bone implants owing to their biocompatibility and osteoinductive ability (Deng et al., 2018; Yu et al., 2020). This inorganic biomaterial has superior bone induction capacities but is limited to the short-term release of the incorporated particles, and it is thus difficult to use it to exert a long-term osteogenic effect (Zhang et al., 2016). Hydrogels could achieve drug encapsulation and sustained release by either chemical adaptability or a hybrid of different polymers (Kluin et al., 2013; Li et al., 2021). Therefore, designing combined organic–inorganic hydrogels, comprising organic polymers and osteoconductive mineralized components, is a potential strategy for the treatment of critical-size segmental bone defects.

**SCHEME 1:**
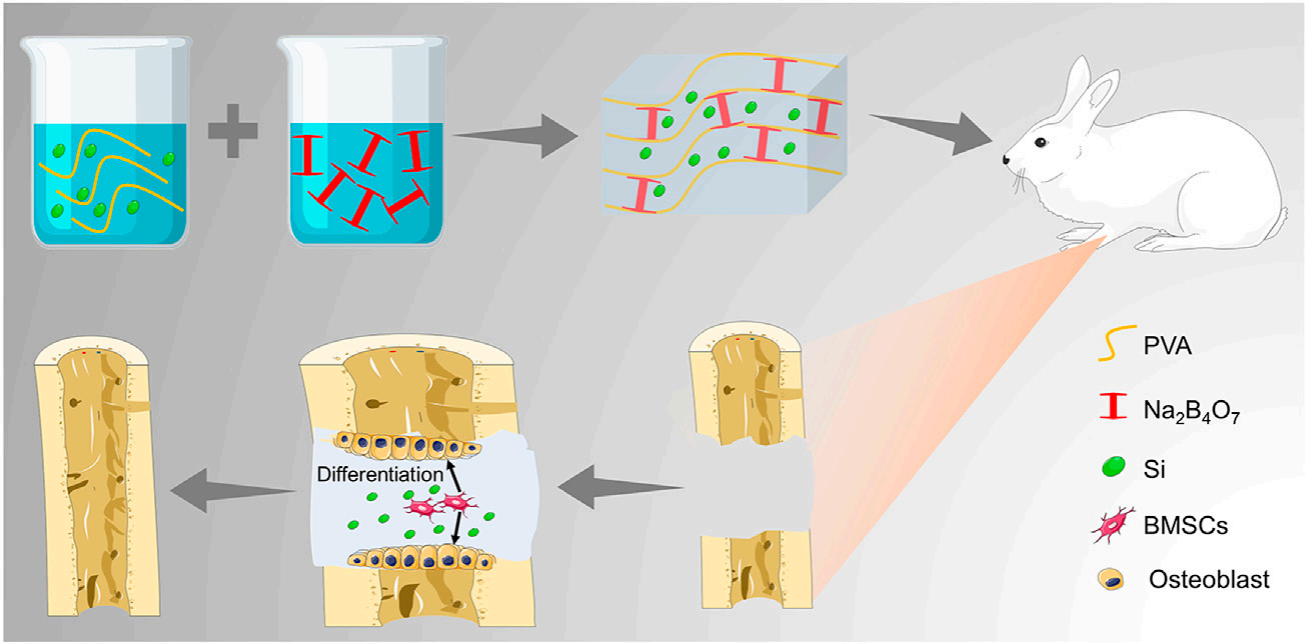
Illustration of the TEOS-hydrogel enhancing critical-size segmental bone defect healing by inducing BMSC osteogenic differentiation.

In this study, we developed a novel multifunctional hydrogel to improve the healing efficiency of segmental bone defects (Scheme 1). The hydrogel was prepared by dynamic supramolecular assembly of polyvinyl alcohol (PVA), sodium tetraborate (Na_2_B_4_O_7_), and tetraethyl orthosilicate (TEOS). In addition to encapsulated osteogenic active ingredients (TEOS), the reversible networks between Na_2_B_4_O_7_ and PVA form the backbone networks of hydrogels. The hydrogels are generated from mainly dynamic supramolecular assembly of Na_2_B_4_O_7_ and PVA that is attributed to the dynamic reactions of PVA–borax–PVA. Self-healing hydrogels maintain affluent adaptable linkages which can be decomposed and are automatically re-combined in a dynamic convertible way, minimizing the adverse influence on the nearby tissue and sustaining their long-term integrality (Zhang and Khademhosseini, 2017). At the same time, self-healing hydrogels supply a permissive microenvironment for the delivery of the substance. The abovementioned advantages cause this self-healing hydrogel to be favorable for multifunctional ingredient-loading in bone defect treatment. The goal of this research is to evaluate the function of this composite hydrogel in inducing osteogenesis in the critical-size radius bone defect rabbit models. Thus, the study will provide a novel strategy for developing bone substitutes in segmental bone defects.

## Materials and Methods

### Materials

Sodium tetraborate (Na_2_B_4_O_7_.10H_2_O) was purchased from Beijing Chemical Works (Beijing, China). PVA (≈99% hydrolyzed, Mw ≈130,000) and TEOS were obtained from Sigma-Aldrich. The rabbit BMSCs were supplied by American Type Culture Collection (ATCC, MD, United States). Low-glucose Dulbecco’s modified Eagle’s medium (LG-DMEM), fetal bovine serum (FBS), and streptomycin–penicillin were obtained from Gibco^®^ Life Technologies (CA, United States). The Cell Counting Kit-8 (CCK-8) kit and Calcein-AM/PI staining kit were supplied by Beyotime Biotechnology (Shanghai, China). The mediums for osteogenic differentiation of BMSCs and Alizarin Red stain were obtained from Cyagen Biosciences (CA, United States). Phosphate buffer (PBS) and 4% paraformaldehyde were provided by Solarbio (Beijing, China). The Eastep Super Total RNA Extraction Kit was purchased from Promega (Shanghai, China), and the Perfect Real-Time RT reagent kit was supplied by Takara Bio (Dalian, China). Hematoxylin–eosin stain was obtained from Thermo Fisher Scientific, MA, United States. Primary antibodies, runt-related transcription factor-2 (Runx-2), type I collagen (Col-1), osteocalcin (OCN), and osteopontin (OPN) were purchased from Abcam (Cambridge, United Kingdom), and secondary antibodies were obtained from Jackson ImmunoResearch Laboratories (West Grove, PA, United States).

### Preparation of Hydrogels

The multifunctional hydrogel was manufactured by the listed protocols. Different concentration of PVA was dissolved in 10 ml of deionized water with magnetic stirring at 90°C. Subsequently, 0.4 mmol TEOS was supplemented into the abovementioned solution. The mixture was strongly stirred. After the components are completely dissolved, 10 ml Na_2_B_4_O_7_ solution was added to the mixture under continuous stirring at 90°C for 10 min until the hybrid hydrogel was constructed. The hydrogel consisting of PVA, Na_2_B_4_O_7_, and TEOS was recognized as TEOS@Gel, while the hydrogel consisting of PVA and Na_2_B_4_O_7_ was abbreviated as Gel as a control.

### Characterization of Hydrogels

The rheology of the hydrogels was investigated by a rheometer (Malvern, United Kingdom). The fixture utilized a circular parallel plate, the diameter of that was 10 mm, and the gap was set as 1,000 μm. During the progression of the experiment, to protect the gel from draining out, the hydrogel was covered with a hood to prevent water evaporation. When the hydrogel was tested in the time sweep mode, the frequency of the rheometer was 10 rad/s, and the strain was 0.1%. While in the strain sweep mode, the frequency and the temperature were set as mentioned above, and the strain was set from 1% to 100%. The morphology of the TEOS hydrogel was observed by field emission scanning electron microscopy (FE-SEM) (JSM-7000F, JEOL, Tokyo, Japan) at 3 kV after freeze-drying, and the obtained images were further analyzed by Image J software (NIH, MD, United States).

The equilibrium cumulative ratio was evaluated by measuring the variation in wet weight by incubating the hydrogels in PBS. In brief, a certain amount of hydrogel (1 g) was weighed and added to PBS, and the quantity of the initial wet hydrogel was noted as W_0_. At predetermined intervals, the hydrogels were collected from the PBS, and the weight was regarded as Wt. The swelling rate = W_t_/W_0_ × 100%. The degradation of hydrogels was conducted by studying the variation of the hydrogel in the dry mass mode. Briefly, the lyophilized hydrogel (100 mg) was placed in PBS at 37°C. At predetermined points, the residual hydrogels were gathered, and the mass loss was recorded after lyophilization.

### Biocompatibility of Hydrogels

To investigate the biocompatibility of the prepared hydrogels, the CCK-8 assay and dead/live staining were conducted. BMSCs were seeded at a density of 2 × 10^4^/well in 24-well plates for different treatment processes. The plates coated with Gel and TEOS/Gel were set as the experimental groups and those without the hydrogel were observed as the control group (abbreviated as Ctrl). At the scheduled time points, cell proliferation was detected by a CCK-8 assay. Briefly, 10% CCK-8 solution was added to the samples and then incubated for 2 h at 37 C. The absorbance was measured by a microplate reader (Multiskan EX, Thermo Fisher Scientific, MA, United States) at 450 nm.

The cell viability was investigated by a Calcein-AM/PI kit after 3 days of incubation according to the manufacturer’s instructions. In brief, Calcein-AM and PI were added to the samples and incubated in the dark for 15 min at 4°C. The fluorescent images were observed and photographed by a confocal laser scanning microscope (CLSM, FV1000, Olympus, Japan), and the cell survival rates were analyzed by ImageJ software according to the proportion of live cells.

### *In Vitro* Osteogenic Induction of Hydrogels

To evaluate the osteogenic differentiation ability of the TEOS-hydrogel, BMSCs were seeded into the 24-well plates at a density of 5 × 10^4^ cells/well. After incubation for 24 h, the medium was changed by the osteogenic induction medium, including LG-DMEM along with β-glycerol-phosphate (10 mM), ascorbate-2-phosphate (50 μM), and dexamethasone (0.1 μM). After 14 and 21 days of osteogenic induction culture, Alizarin Red staining was performed to evaluate the cell mineralization by observing the calcium nodule deposition. Subsequently, 10% cetylpyridinium chloride was added to the samples to dissolve the mineralized nodules for further semi-quantitative analysis by a microplate reader at 562 nm.

### Real Time-qPCR

The expression of genes involved in osteogenic differentiation was determined by real-time quantitative PCR (RT-qPCR), including *Runx-2*, *Col-1*, *OCN*, and *OPN*, in the BMSCs at 14 and 21 days after osteogenic induction and bone tissues. The primers sequences are listed in Table 1. Total RNA was collected by TRIzol reagent. Synthesis of cDNA was performed using an Eastep Super Total RNA Extraction Kit according to the manufacturer’s instructions. An A260/A280 value of approximate 2.0 was generally accepted for further analysis. The amplification and performance of RT-qPCR were carried out by adopting 2× Fast SYBR Green Master Mix (Roche Diagnostics, Basel, Switzerland) and detected by a LightCycler 480 (Roche Diagnostics). The relative mRNA expression was normalized by GAPDH and calculated according to the formula of the 2^−ΔΔCT^ method. For RT-qPCR of the bone, tissues were collected and quickly placed in a mortar precooled with liquid nitrogen and repeatedly ground to powder in liquid nitrogen for RT-qPCR as previously described.

**TABLE 1:** Primer sequences of genes.

Gene	Oligonucleotide primers (5′-3′)
*Runx-2*	F: 5′-ACT​ACC​AGC​CAC​CGA​GAC​CA-3′
	R: 5′-ACT​GCT​TGC​AGC​CTT​AAA​TGA​CTC​T-3′
*Col-1*	F: 5′-TCC​GGC​TCC​TGC​TCC​TCT​TA-3′
	R: 5′-GGC​CAG​TGT​CTC​CCT​TG-3′
*OCN*	F: 5′-AGC​CAC​CGA​GAC​ACC​ATG​AGA-3′
	R: 5′-AGC​CAC​CGA​GAC​ACC​ATG​AGA-3′
*OPN*	F: 5′-GCT​AAA​CCC​TGA​CCC​ATC​T-3′
	F: 5′-CGT​CGG​ATT​CAT​TGG​AGT-3′
*GAPDH*	F: 5′-CAA​TGA​CCC​CTT​CAT​TGA​CC-3′
	R: 5′-TGG​ACT​CCA​CGA​CGT​ACT​CA-3′

### Immunofluorescence

After 21 days of osteogenic induction culture, immunofluorescence was conducted to investigate the Runx-2, Col-1, OCN, and OPN expression. Briefly, the cell samples were fixed with 4% paraformaldehyde for 20 min and then permeabilized with 0.1% Triton X-100 solution for 5 min. After that, nonspecific binding was blocked with 3% BSA for 30 min. The samples were incubated with primary antibodies, including anti-Runx-2 (1:100), anti-Col-1 (1:250), anti-OCN (1:200), and anti-OPN (1:250) overnight at 4°C. The samples were then washed with PBS three times and treated with the secondary antibody for 5 h. Goat anti-rabbit IgG and goat anti-mouse IgG conjugated to fluorescein isothiocyanate were added at a dilution of 1:200 and incubated. The negative control samples were not subjected to primary antibody incubation. After being washed with PBS three times, the nucleus was stained with DAPI. Finally, the fluorescence intensity of the cells was observed with a CLSM.

### Establishment of Critical-Size Bone Defect Models

A total of 36 New Zealand white rabbits (female, six months old) were selected to prepare the infected radius fracture models. The critical-size segmental radial bone defect models were prepared under general anesthesia with 3% (w/v) pentobarbital at the dose of 50 mg/kg. After preoperative preparation, the left lateral radial incision was selected to expose the tissue layer by layer. When the bony surface of the left radius was exposed, a 15- mm segmental bone defect was prepared by a bone drill. The periosteum around the ends of the defect was excised to prevent ossification. Then, the defects were rinsed, and the animals were divided into three groups, namely, the control group without hydrogel implantation, the Gel group with 500 μl hydrogel, and the TEOS/Gel group with 500 μl TEOS-hydrogel. Finally, the incisions were sutured layer by layer, and the animals were injected intramuscularly with penicillin (1.5 mg/kg) for 3 consecutive days to prevent infection.

At 6 and 12 weeks after surgery, the rabbits were euthanized by intravenous injection of pentobarbital sodium (120 mg/kg). The left radius samples were collected for sequential studies.

### Micro-CT

To evaluate the bone regenerative efficiency, the bone tissues were scanned by Micro-CT (SkyScan 1076 scanner, Bruker, Belgium) in a high-resolution scanning mode with a pixel size of 18 μm. Subsequently, quantitative morphometric analysis of bone volume/tissue volume ratio (BV/TV, %) of the original defect region, regarded as the region of interest, was analyzed by micro-CT auxiliary software (NRecon version 1.6.6).

### Histological Evaluation

All collected radius samples were fixed with 4% paraformaldehyde for 1 week and then decalcified in Morse’s solution for another 5 weeks. The samples were dehydrated in graded ethanol series, paraffin-embedded, and sectioned to 5-μm thickness slices. Hematoxylin and eosin (H&E) staining was conducted to observe new bone formation.

In addition, immunofluorescence staining was performed to detect the expression of Runx-2, Col-1, OCN, and OPN in the regenerative tissues. Briefly, the slices were washed with PBS and blocked with 3% BSA in PBS containing 0.2% Triton X-100 for 60 min. Subsequently, the slices were incubated with primary antibodies, 1:200 Runx-2 anti-mouse polyclonal antibody along with 1:250 Col-1 anti-rabbit monoclonal antibody and 1:150 OCN anti-mouse polyclonal antibody along with 1:200 OPN anti-rabbit polyclonal antibody overnight at 4°C. After washing with PBS three times, the slices were then incubated with 1:600 Cy3-conjugated goat anti-rabbit or 1:800 goat anti-mouse IgG DyLight 488-conjugated secondary antibodies for 1 h at 37°C. The nuclei were stained with DAPI (1:600) and observed by CLSM.

### Statistical Analysis

All results were calculated as mean ± standard deviation (SD). Comparisons among groups were analyzed with one-way ANOVA followed by Tukey’s post hoc test using SPSS 19.0 (SPSS Inc., Chicago, IL, United States). *P* < 0.05 was considered statistically significant.

## Results and Discussion

### Preparation and Characterization of Hydrogels

**FIGURE 1:**
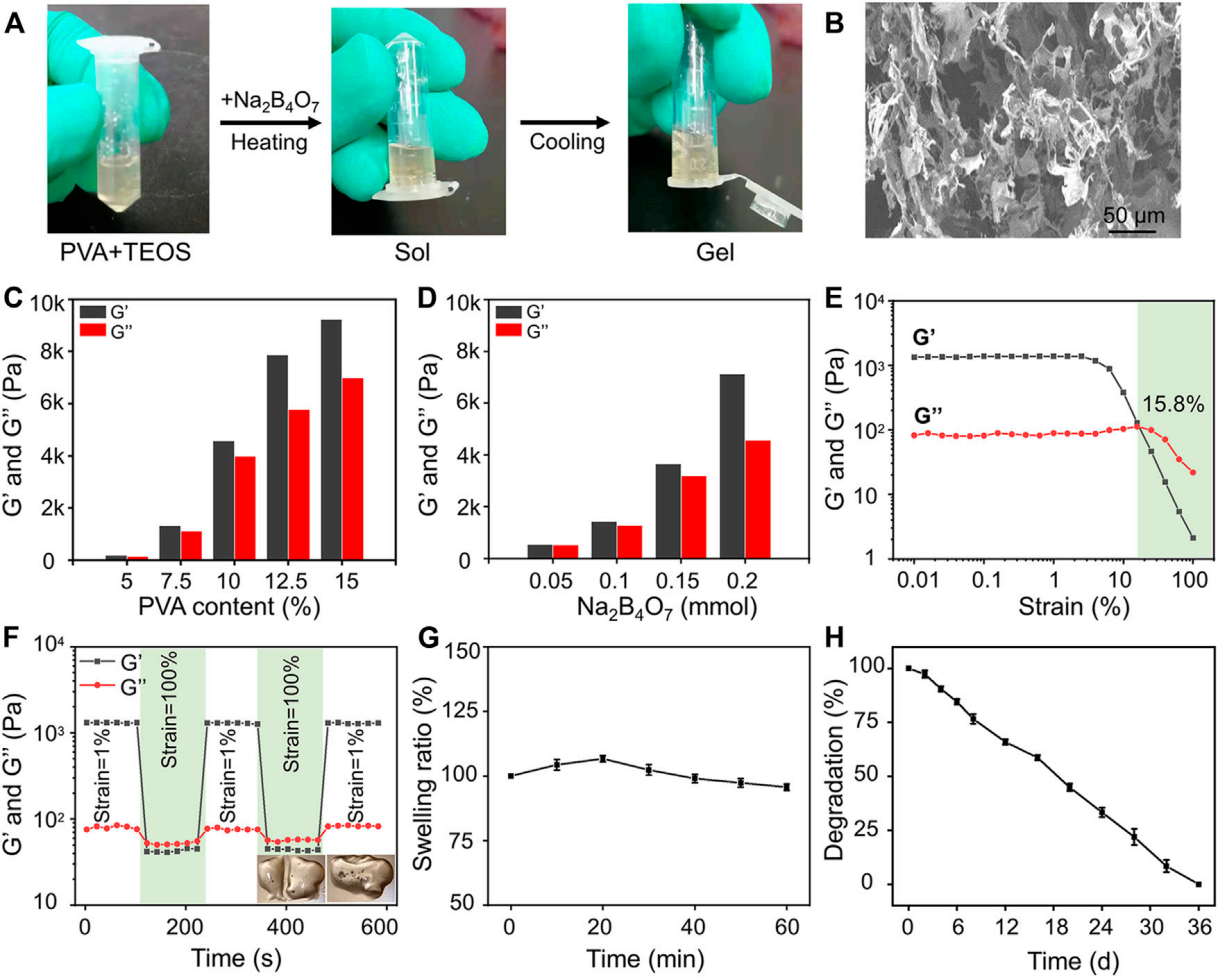
Preparation and characterization of hydrogels. **(A)** Photographs of the gelation progress. The mixture of the prepared PVA and TEOS solution was presented in the sol state, and it experienced sol–gel transition after adding Na_2_B_4_O_7_ solution. **(B)** SEM observation of prepared hydrogels. **(C)** Storage modulus and loss modulus of prepared hydrogels as the concentration of PVA increased from 5 to 15 wt%. **(D)** Storage modulus and loss modulus of prepared hydrogels as the concentration of Na_2_B_4_O_7_ increased from 0.05 to 0.20 mmol. **(E)** Strain amplitude sweep of the hydrogel. **(F)** Step strain measurements of the hydrogel with a fixed frequency of 1 rad/s, and the macroscopic observation of self-healing property. **(G)** Equilibrium swelling ratio. **(H)** *In vitro* degradation (the tested hydrogel formula is 10 wt% PVA, 0.20 mmol Na_2_B_4_O_7_, 0.4 mmol TEOS).

The preparation strategy of the hydrogels is shown in Figure 1A. Completely dissolved PVA and TEOS were added into the Na_2_B_4_O_7_ solution at 90°C. Subsequently, the temperature was decreased to 25°C to complete the sol–gel transition rapidly. The hydrogels were designed by dynamic supramolecular assembly of Na_2_B_4_O_7_, PVA, and TEOS. The microstructures of the prepared hydrogels were observed by SEM, and the images showed that the lyophilized hydrogels had an interconnected porous structure (Figure 1B). These interconnected nanochannels benefit oxygen and nutrient transport, thus promoting cell communication and improving cell survival, which is valuable for biological applications (Kumar et al., 2018).

The mechanical properties of hydrogels can be adjusted by changing the proportion of crosslinking components. The gel dynamics of hydrogels with different concentrations were monitored by recording the storage modulus (G′) and loss modulus (G″) in the time scan mode. According to the rheological analysis, the results indicated that along with the PVA and Na_2_B_4_O_7_ content increase, the G′ and G″ of the hydrogels increased correspondingly (Figures 1C,D). On the contrary, the concentration of TEOS had little effect on the dynamic properties of the hydrogels. As shown in Figure 1E, rheological performances of the prepared hydrogels were detected under the strain scan mode (0.01%–100%). In the low-strain area, both G′ and G″ are maintained at a constant value. Along with the strain increase, the G′ and G″ curves intersected at a strain of 15.8%, which was the required critical strain value to destruct the hydrogel network. In addition, based on the strain amplitude scanning results, the self-healing property of hydrogels was observed by continuous variation of shear strains (1% and 100%). As exhibited in Figure 1F, G″ was higher than G′ at higher shear strains (100%) indicating that the hydrogels were destroyed. Soon afterward, when the shear strain dropped to 1%, G′ and G″ almost completely recovered to the initial value, showing that the hydrogel network structure was restored. This repeatable process proved that this hydrogel has a durable self-healing property. For macroscopic observation, the hydrogels were physically cut into two halves, and two separate hydrogels can be reassembled into one whole in 30 min, displayed in the lower right of Figure 1F. This excellent self-healing feature is attributed to the dynamic characteristics of boronate formation between the Na_2_B_4_O_7_ and PVA. Self-healing hydrogels have affluent adaptable linkages that can be broken and reconnect automatically in the form of dynamic reversibility to minimize adverse effects on the surrounding tissue and maintain their long-term integrity.

Then, the swelling performance of the hydrogels was investigated in PBS and shown in Figure 1G. The hydrogels reached swelling equilibrium at about 20 min, and the maximum swelling rate of the hydrogels was about 7%. Moreover, in the process of the swelling test, the hydrogels maintained its initial shape without destruction. This physical and chemical stability made the hydrogels an ideal implant for *in vivo* application. The degradation property of materials is also another critical factor for transplantation. According to the measurement of the change of dry weight of the hydrogels, we found that the hydrogels could degrade completely within 36 days (Figure 1H).

### Biocompatibility of Hydrogels

**FIGURE 2:**
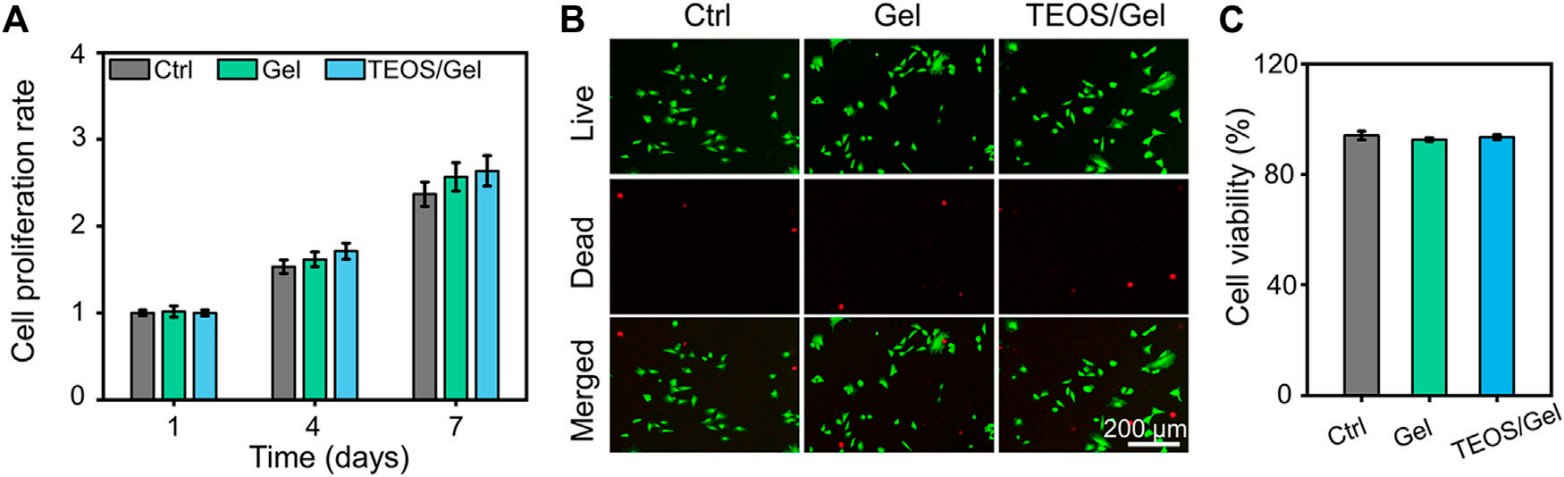
Biocompatibility of the prepared hydrogels. **(A)** Cell proliferation rate at days 1, 4, and 7. **(B)** Calcein AM/PI staining of BMSCs at day 3. **(C)** Quantitative analysis of the cell survival rate by Calcein AM/PI staining.

The biocompatibility of hydrogels is the prerequisite for their biomedical applications. The cell proliferation and viability of BMSCs cultured in the hydrogels were revealed by CCK-8 detection and Calcein-AM/PI staining. The results indicated that BMSCs in the control, Gel, and TEOS/Gel groups proliferated gradually without statistical difference among the groups on days 4 and 7 (Figure 2A), indicating that the TEOS hydrogel did not have any negative effect on the proliferation of BMSCs. The pictures of Calcein AM/PI staining demonstrated that the BMSCs maintained good cell viability in all groups on day 3 (Figure 2B). Furthermore, the cell survival rate of BMSCs in the control, Gel, and TEOS/Gel groups was 94.1 ± 1.6%, 92.6 ± 0.7%, and 93.5 ± 0.9%, respectively, and there was no significant difference among the groups (Figure 2C). The abovementioned results demonstrated that our hydrogels had good biocompatibility for BMSCs and had wide application prospects in tissue engineering.

### Tetraethyl Orthosilicate Hydrogel Induced Bone Marrow Mesenchymal Stem Cell Osteogenic Differentiation

**FIGURE 3:**
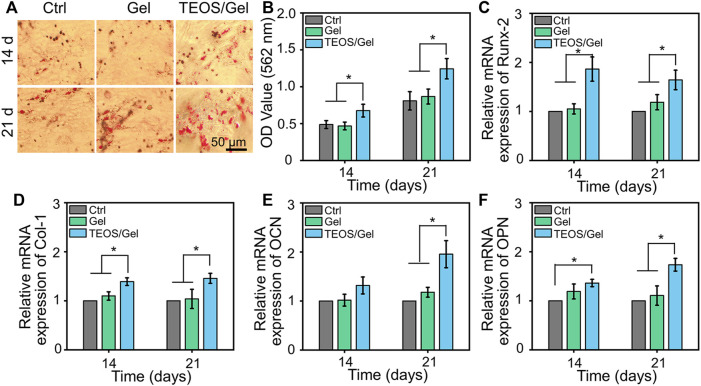
TEOS hydrogel induces BMSC osteogenic differentiation. **(A)** Gross images of Alizarin Red staining. **(B)** Semi-quantitative analysis of Alizarin Red staining. **(C–F)** mRNA expression levels of osteogenic-related genes, including *Runx-2*, *Col-1*, *OCN*, and *OPN*, in BMSCs after osteogenic induction for 14 and 21 days (*p < 0.05).

In addition to the biocompatibility of the materials, the capacity of inducing BMSC osteogenic differentiation is a crucial factor for the initiation of bone formation. The deposition of calcium nodules is a hallmark event of osteogenic differentiation of BMSCs, which can be observed by Alizarin Red dye (Bai et al., 2020a). As indicated in Figure 3A, gross images showed that more calcium nodules were observed in the TEOS/Gel group than in the control group and Gel group on the 14th and 21st days. More precisely, semi-quantitative analysis was used to further demonstrate that the absorbance in the TEOS/Gel group was significantly higher than that in the control group and Gel group (*p* < 0.05, Figure 3B). These results indicated that the TEOS hydrogel enhanced the deposition of mineralized nodules. In addition, to investigate the influence of the TEOS hydrogel on osteogenic differentiation at the gene level, the expression of some crucial osteogenic-related genes, including *Runx-2*, *Col-1*, *OCN*, and *OPN*, was analyzed by RT-qPCR. The upregulation of *Runx-2* expression is a key event in the early osteogenic differentiation process, which can activate the expression of *OCN* and *OPN* (Sivashanmugam et al., 2017). As displayed in Figure 3C, the expression of *Runx-2* in the TEOS/Gel group was 1.9-fold and 1.8-fold on the 14th day and 1.6-fold and 1.4-fold on the 21st day, higher than that in the control group and Gel group, respectively (*p* < 0.05). Similarly, the expression of *Col-1*, *OCN*, and *OPN* in the TEOS/Gel group was significantly upregulated than that in the control group and Gel group (Figures 3D–F).

**FIGURE 4:**
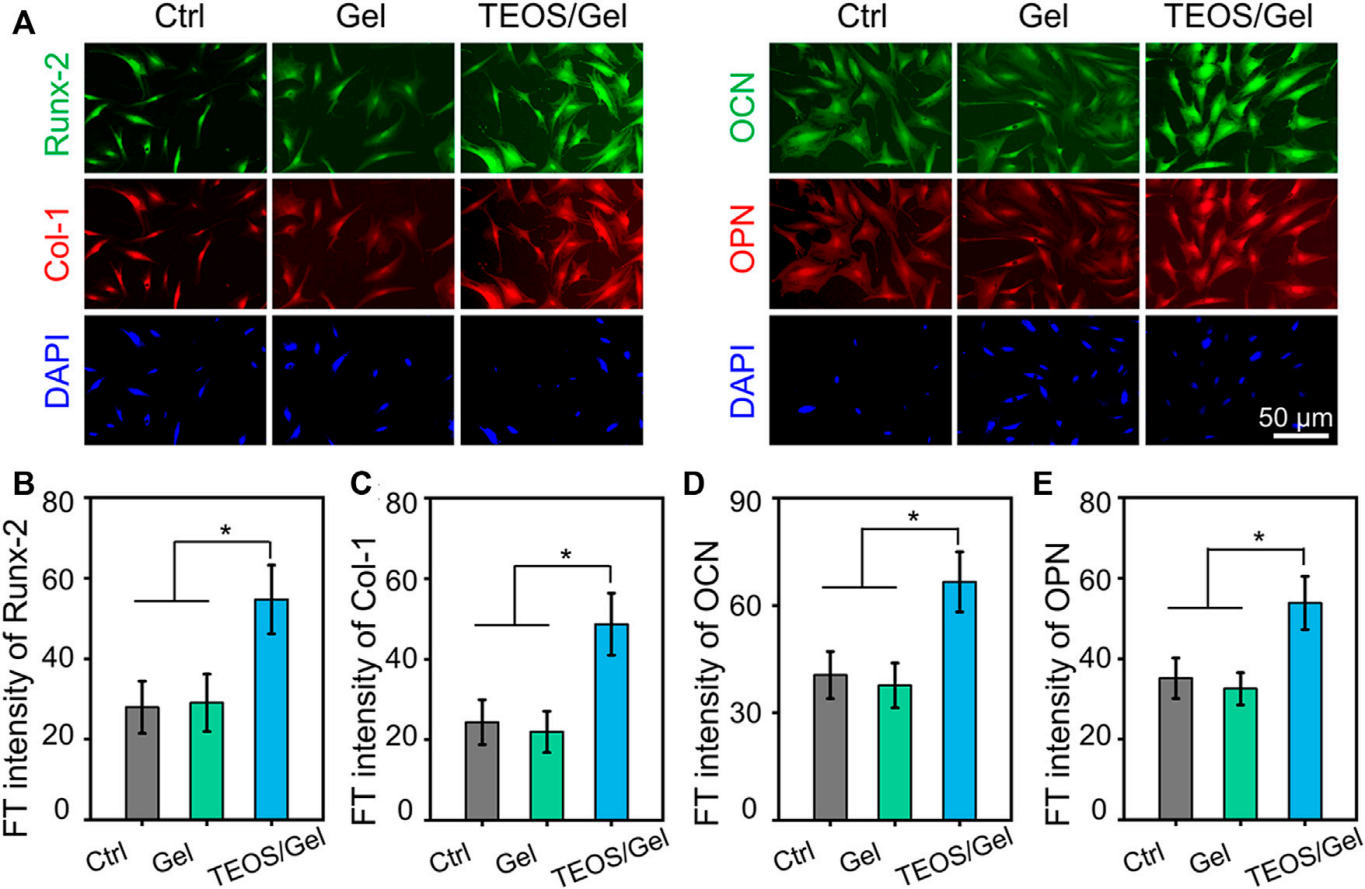
TEOS hydrogel induces BMSC osteogenic differentiation at the protein level. **(A)** Double immunofluorescent staining of cartilage-specific markers Runx-2, Col-1, OCN, and OPN in BMSCs after osteogenic induction for 21 days. **(B–E)** Quantitative analysis of the immunofluorescence intensity of Runx-2, Col-1, OCN, and OPN (*p < 0.05).

In addition, to investigate the function of the TEOS hydrogel on the expression of osteogenic differentiation at the protein level, protein markers (Runx-2, Col-1, OCN, and OPN) were stained by double immunofluorescence after 21 days of osteogenic induction culture (Figure 4A). As shown inFigures 4B–E, quantitative analysis of the fluorescence of osteogenic-related markers revealed that the intensity in the TEOS/Gel group was significantly enhanced than that of the control group (*p* < 0.05) and Gel group (*p* < 0.05).

Owing to the positive role of silicon in inducing bone regeneration, various silicon-based bone substitutes (such as nano silicates, silicon dioxide, etc.) have been widely studied and developed as potential orthopedic implants (Carlisle, 1970). It is of importance that a series of studies have demonstrated that the bioactive Si ions can enhance cell proliferation and promote stem cell osteogenic differentiation (Wang et al., 2013). Incorporating Si ions into bone tissue engineering biomaterials significantly upregulated angiogenic factor expression and induced osteogenic differentiation of BMSCs (Sun et al., 2018). Herein, the TEOS hydrogel was prepared, and the osteogenic induction capacity was investigated. The results demonstrated that our TEOS hydrogel could significantly enhance calcium nodule deposition and upregulate osteogenesis-related marker expression, thus inducing BMSC osteogenic differentiation.

### Tetraethyl Orthosilicate Hydrogel Promoted Critical-Size Segmental Bone Defect Repair

**FIGURE 5:**
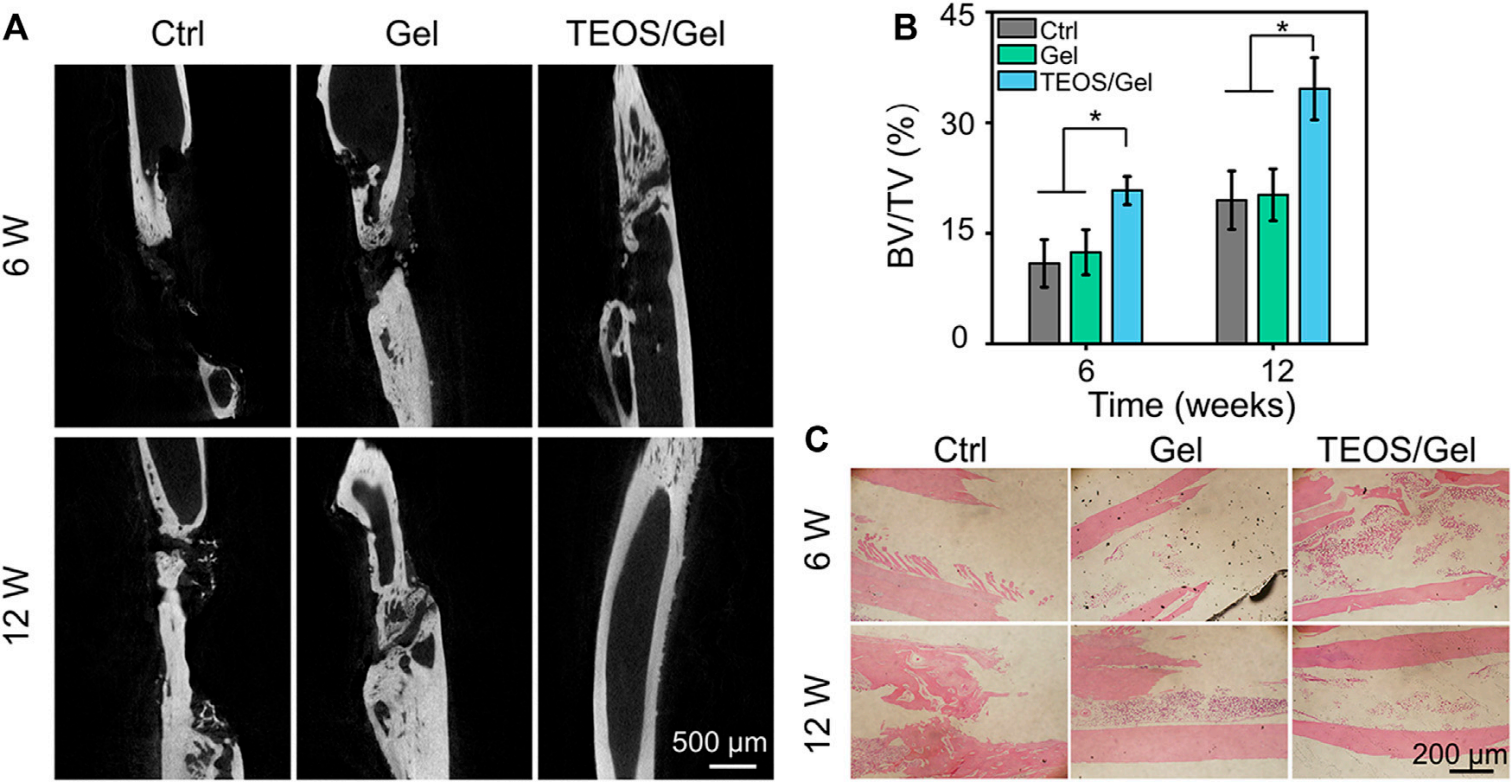
TEOS hydrogel promoted critical-size segmental bone defect repair. **(A)** Micro-CT images of regenerated bone tissue at the 6th and 12th week after transplantation. **(B)** Quantitative analysis of BV/TV of the regenerated bone in the defects according to micro-CT. **(C)** H&E staining for histological observation of radius critical-size defects (*p < 0.05).

Severe trauma, bone tumors, congenital malformation, or extensive infection of bone tissue can be the causes of critical-size bone defects. A critical-size bone defect is defined as an orthotropic intraosseous wound that does not self-heal without additional surgical intervention (Van Houdt et al., 2021). The repair of critical-size segmental bone defects is still a great challenge in clinic. Over the past decades, different types of synthetic bone grafts have been developed, of which particularly calcium phosphate (CaP) and hydroxyapatite-based bone substitutes have shown great potential (Zhao R. et al., 2020; Zhao Y. et al., 2020; Chae et al., 2021; Liu et al., 2021; He et al., 2022). In view of increased demands for synthetic bone grafts with advantageous properties, we designed a self-healing hydrogel consisting of PVA, Na_2_B_4_O_7_, and TEOS. In this study, micro-CT was conducted during the 6th and 12th weeks after surgery to investigate the efficacy of the TEOS hydrogel on bone healing in critical-size bone defects. As displayed in Figure 5A, the TEOS hydrogel induced bone healing and restored the defects in a time-dependent manner. Obvious bone regeneration was observed at the 6th week, and the defects almost healed at the 12th week after transplantation in the TEOS/Gel group. However, in the control group and Gel group, limited bone regeneration, bone discontinuity, and non-union can be observed at the defects. To further quantify bone repair in the defects, the area of critical-size segmental bone defects was regarded as the region of interest to analyze the regenerated bone mass by Micro-CT software. The bone volume/total volume (BV/TV) values of the control, Gel, and TEOS/Gel groups were 10.9 ± 3.2%, 12.4 ± 3.0%, and 20.8 ± 1.9% during the 6th week and 19.5 ± 3.9%, 20.2 ± 3.5%, and 34.6 ± 4.2% during the 12th week, respectively (Figure 5B). Furthermore, H&E staining was performed to further observe the regenerated bone. As exhibited in Figure 5C, the histological observation was consistent with that of Micro-CT analysis, which indicated that the absence of bony bridge formation was in the area of the defects in the control group and Gel group. Moreover, the TEOS/Gel transplantation achieved satisfactory bone repair effect and recanalization of the medullary cavity. The abovementioned radiological evaluation and histological observation indicated that the critical-size segmental bone defects were difficult to self-regenerate effectively without additional treatment intervention, but our TEOS hydrogel with fascinating osteogenic induction function can significantly promote bone repair.

### Tetraethyl Orthosilicate Hydrogel Enhanced Osteogenic-Related Marker Expression *In Vivo*

**FIGURE 6:**
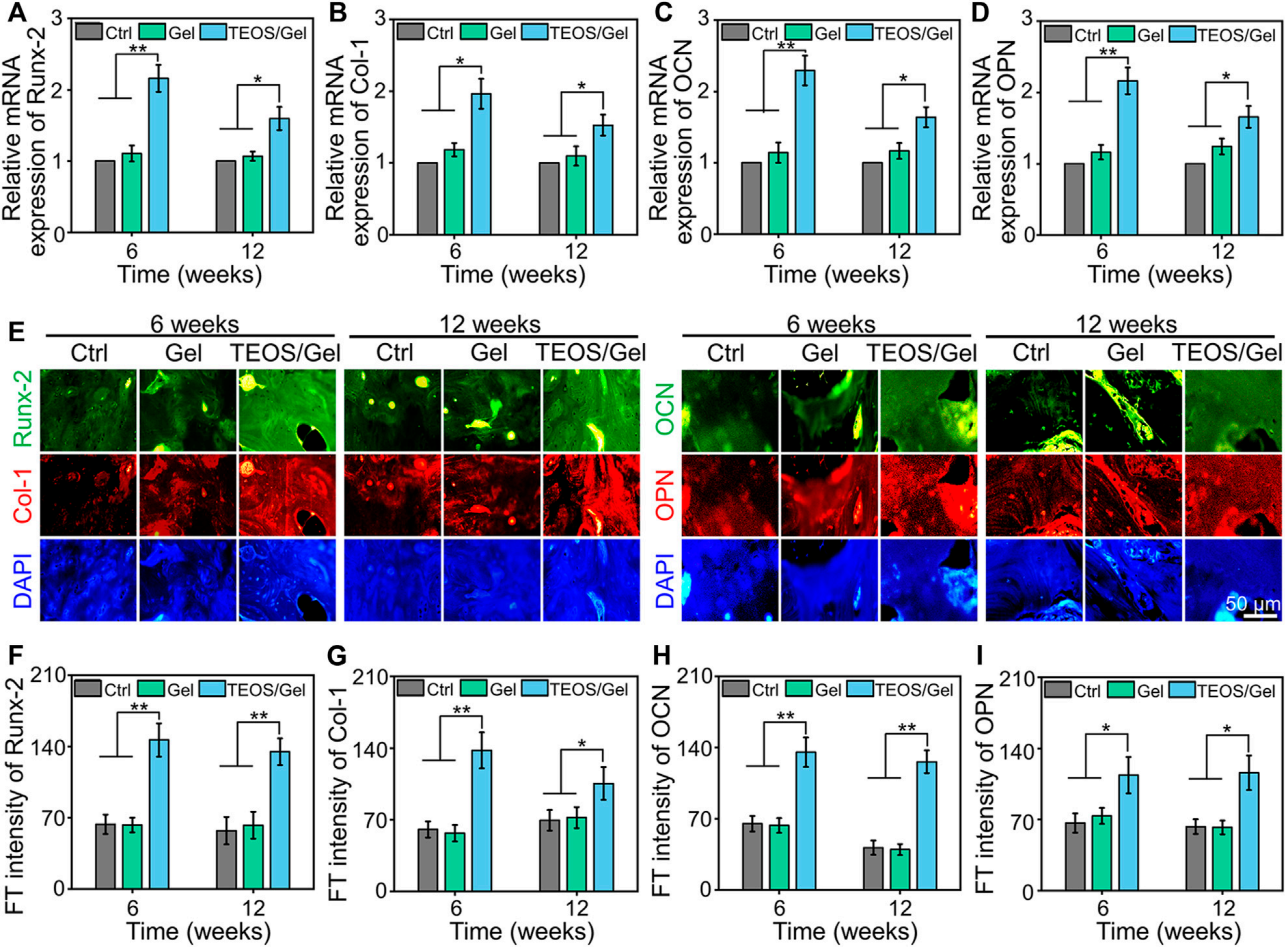
TEOS hydrogel enhanced osteogenic-related marker expression. **(A–D)** RT-qPCR analysis of *Runx-2*, *Col-1*, *OCN*, and *OPN* in the regenerated bone tissues. **(E)** Representative immunofluorescence images. **(F–I)** Quantitative analysis of fluorescence intensity of Runx-2, Col-1, OCN, and OPN in the regenerated bone tissues (*p < 0.05; **p <0.01).

To investigate the *in vivo* mechanism of the TEOS hydrogel promoting bone healing, we collected bone tissue for RT-qPCR detection and immunofluorescence of osteogenesis-related markers. Runx-2 is a member of the Runx family of transcription factors and is also one of the earliest indications of osteoblastic differentiation. Runx-2 can induce the expression of key osteogenic genes such as OCN and OPN (Bai et al., 2020b). Col-1 is another critical osteogenic differentiation gene. The reticular structure formed by Col-1 is not only the premise of realizing the mineralization function of osteoblasts but also an important index reflecting the bone formation ability (Zhao Y. et al., 2020). OCN and OPN are bone-specific proteins synthesized by osteoblasts and recognized as a marker to assess osteogenic maturation and bone formation, and it shows the highest level during the late stage of osteogenesis (Qiao Y. et al., 2020; Kim et al., 2021). The expression of *Runx-2*, *Col-1*, *OCN*, and *OPN* at the transcriptional level in the TEOS/Gel group was significantly upregulated compared with that of the control group and Gel group in the regenerated bone tissues (Figures 6A–D). Moreover, immunofluorescence was performed to further reveal the expression of osteogenic differentiation markers in the regenerated bone tissues at the translation level (Figure 6E). Quantitative analysis of the immunofluorescence images indicated that the fluorescence intensity of Runx-2, Col-1, OCN, and OPN in each group had the same trend as the expression at the gene level (Figures 6F–I). These abovementioned results demonstrated that transplantation of the TEOS hydrogel to the critical-size segmental bone defects can promote the upregulation of osteogenic markers.

In general, materials with osteogenic induction capacity are considered potential complementary components in implants to obtain better bone regeneration outcomes (Huang et al., 2016). Silicon is one of the essential trace elements in the human body, which shows a critical role in bone development and repair. Si is widely distributed in organelles such as mitochondria and acts as a cross-linking agent for collagen and proteoglycans, thus inducing Col-1 secretion and enhancing mineralization in the early stage of bone regeneration (He et al., 2018). In addition, Si ions can regulate the interaction between cells and induce stem cell migration and differentiation to promote osteogenesis and angiogenesis (Wang et al., 2018). Taking into account the critical function of Si in bone regeneration, the application of Si in hydrogel design is highly valued. In our study, the silicon-containing TEOS hydrogel can induce osteogenic differentiation of BMSCs and promote critical-size segmental bone defect repair *in vivo* by upregulating osteogenic-related markers, including Runx-2, Col-1, OCN, and OPN.

## Conclusion

We prepared a functional hydrogel with satisfactory osteogenic effects through the dynamic supramolecular assembly of PVA, Na_2_B_4_O_7_, and TEOS, as bone substitutes. This TEOS hydrogel has adjustable mechanical properties, self-healing capacity, degradability, and biocompatibility, and it promotes BMSC osteogenic differentiation. Above all, the TEOS hydrogel significantly promotes bone healing in critical-size segmental bone defects. Therefore, it is anticipated that the TEOS hydrogel may have potential applications in the management of critical-size segmental bone defects.

## Data Availability

The original contributions presented in the study are included in the article/Supplementary Material; further inquiries can be directed to the corresponding authors.
